# Effect of efflux pump inhibition on *Pseudomonas aeruginosa* transcriptome and virulence

**DOI:** 10.1038/s41598-017-11892-9

**Published:** 2017-09-12

**Authors:** Giordano Rampioni, Cejoice Ramachandran Pillai, Francesca Longo, Roslen Bondì, Valerio Baldelli, Marco Messina, Francesco Imperi, Paolo Visca, Livia Leoni

**Affiliations:** 1 Department of Science, University Roma Tre, Rome, Italy; 2 Department of Biology and Biotechnology “Charles Darwin”, Sapienza University of Rome, Rome, Laboratory affiliated to Istituto Pasteur Italia – Fondazione Cenci Bolognetti, Rome, Italy; 3 Present Address: Inter University Centre for Bioscience, Kannur University, Palayad, Kerala India

## Abstract

Efflux pumps of the resistance-nodulation-cell-division (RND) family increase antibiotic resistance in many bacterial pathogens, representing candidate targets for the development of antibiotic adjuvants. RND pumps have also been proposed to contribute to bacterial infection, implying that efflux pump inhibitors (EPIs) could also act as anti-virulence drugs. Nevertheless, EPIs are usually investigated only for their properties as antibiotic adjuvants, while their potential anti-virulence activity is seldom taken into account. In this study it is shown that RND efflux pumps contribute to *Pseudomonas aeruginosa* PAO1 pathogenicity in an insect model of infection, and that the well-characterized EPI Phe-Arg-β-naphthylamide (PAβN) is able to reduce *in vivo* virulence of the *P*. *aeruginosa* PAO1 laboratory strain, as well as of clinical isolates. The production of quorum sensing (QS) molecules and of QS-dependent virulence phenotypes is differentially affected by PAβN, depending on the strain. Transcriptomic and phenotypic analyses showed that the protection exerted by PAβN from *P*. *aeruginosa* PAO1 infection *in vivo* correlates with the down-regulation of key virulence genes (*e*.*g*. genes involved in iron and phosphate starvation). Since PAβN impacts *P*. *aeruginosa* virulence, anti-virulence properties of EPIs are worthy to be explored, taking into account possible strain-specificity of their activity.

## Introduction

Introduction of any antibiotic in the clinical practice invariably results in ensuing resistance. The indiscriminate use of antibiotics and the increasing emergence of antibiotic resistance has drained the research in this field, resulting in a discovery rate of new antibiotics unable to compensate the escalation of antibiotic resistance in common pathogens^1, 2^.

The serious economic and health problems caused by multi-drug resistant (MDR) pathogens have fostered research not only into new antibiotics but also into novel adjuvants^1, 2^. Different from conventional antibiotics, adjuvants share the distinctive feature of targeting bacterial factors not essential for growth, such as virulence determinants (*e*.*g*. toxins, adhesins and tissue-degrading enzymes) or antibiotic resistance determinants (*e*.*g*. efflux pumps, antibiotic inactivating enzymes). Such treatments are aimed at facilitating host immune response and/or antibiotic action in clearing the infection. As to anti-virulence drugs, they are predicted to exert a low selective pressure for the emergence of resistant strains, since they do not directly inhibit bacterial growth^2–4^.

The active efflux of antibiotics *via* efflux pumps contributes to the bacterial MDR phenotype, and the development of efflux pump inhibitors (EPIs) is considered a promising adjuvant strategy^2, 5–7^. Efflux pumps are categorized into different families on the basis of the amino acid sequence, the energy source required to drive antibiotic export, and the substrate specificity. The resistance-nodulation-cell-division (RND) family of efflux pumps is considered a viable target for the development of drugs aimed at increasing bacterial susceptibility to antibiotics, due to their prominent contribution to the MDR phenotype and to the absence of human homologues^2, 5–7^. Notably, evidence is emerging that some RND transporters are also involved in the efflux of bacterial factors important for virulence^8, 9^. These preliminary observations suggest that EPIs targeting RND efflux pumps could also affect bacterial virulence, in addition to facilitating antibiotic activity.

*Pseudomonas aeruginosa* is one of the most dreaded opportunistic pathogens, representing a paradigm of Gram-negative MDR “superbug” for which effective therapeutic options are limited. The ability of *P*. *aeruginosa* to cause a wide range of infections in humans is due to its capacity to produce a large repertoire of virulence factors and, ultimately, respond and adapt to harsh conditions as those imposed by the host immune response and antibiotic exposure^1^. The pathogenic potential of *P*. *aeruginosa* relies on the coordinated expression of a large array of virulence factors, the majority of which are positively controlled by quorum sensing (QS)^10^. The three main *P*. *aeruginosa* QS systems are based on the production of specific signal molecules, namely the *N*-acyl-homoserine lactones (AHLs) *N*-3-oxododecanoyl-homoserine lactone (3OC_12_-HSL) and *N*-butanoyl-homoserine lactone (C_4_-HSL), and the 2-alkyl-4-quinolones (AQs) 2-heptyl-4-hydroxyquinoline (HHQ) and 2-heptyl-3-hydroxy-4-quinolone (PQS). These systems are hierarchically organized, since 3OC_12_-HSL is required for optimal production of all QS signals^10^. Moreover, the *P*. *aeruginosa* genome is predicted to encode multiple RND efflux pumps, four of which are of clinical importance for MDR, namely MexAB-OprM, MexCD-OprJ, MexEF-OprN and MexXY-OprM, and are frequently found to be up-regulated in clinical isolates^11^.

The MexAB-OprM is considered as the most important RND efflux pump for *P*. *aeruginosa*, since it is constitutively expressed and provides intrinsic resistance to a broad spectrum of antibiotics^11^. The emergence of *P*. *aeruginosa* MexAB-OprM over-expressing mutants in a rat model of acute pneumonia suggests that this efflux pump confers a selective advantage *in vivo*, also in the absence of antibiotic treatment^12^. Moreover, *P*. *aeruginosa* lacking the MexAB-OprM efflux pump could not invade Madin-Darby canine kidney (MDCK) epithelial cells, and invasion could be restored by supplementation with culture supernatants obtained from MDCK cells infected with wild type *P*. *aeruginosa* ^13^. In addition, it was reported that MexAB-OprM participates in the efflux of 3OC_12_-HSL^14, 15^ and that MexEF-OprN and MexGHI-OprM could be involved in transport of some AQs^16, 17^. All these data argue for a role of MexAB-OprM and other *P*. *aeruginosa* RND efflux pumps in the export of virulence determinants contributing to invasiveness and infection.

Phe-Arg-β-naphthylamide (PAβN, also named MC-207,110) is the most active and best studied inhibitor of *P*. *aeruginosa* RND efflux pumps. It was discovered in a screen for adjuvants of the fluoroquinolone levofloxacin, carried out in a *P*. *aeruginosa* strain that over-expressed MexAB-OprM, though this EPI was also found to be active against other RND pumps like MexCD-OprJ and MexEF-OprN^18, 19^, indicating that PAβN is a broad spectrum EPI^2, 5^. In agreement with the results obtained with MexAB-OprM-deficient cells^13^, it has been shown that PAβN reduces the invasiveness of *P*. *aeruginosa* in MDCK cells^20^, suggesting that this compound could also inhibit some *P*. *aeruginosa* virulence traits. Indeed, PAβN decreases the production of the QS signals 3OC_12_-HSL and C_4_-HSL, and of some QS-dependent virulence phenotypes in *P*. *aeruginosa* MDR isolates from urinary and wound infections^21^. Beside its role as EPI, it has been reported that PAβN can affect *P*. *aeruginosa* membrane permeability, and consequently bacterial growth, when used beyond certain concentrations (~50–200 µM)^19, 22^. This side effect is particularly relevant in efflux pumps deficient genetic backgrounds^19, 22^, and complicates the understanding of the mechanism of action of PAβN as an EPI and as a virulence inhibitor.

This study is aimed at investigating the effect of PAβN on the general physiology and virulence of the widely studied model strain *P*. *aeruginosa* PAO1, by performing microarray analysis and *Galleria mellonella* infection experiments. We also provide evidence that PAβN affects to different extent virulence-related phenotypes in *P*. *aeruginosa* clinical isolates.

## Results and Discussion

### PAβN treatment extensively affects the *P*. *aeruginosa* transcriptome

A major requirement for anti-virulence drugs is their ability to inhibit virulence traits without affecting cell viability^3, 4^. Hence, PAβN concentrations not affecting the growth rate of *P*. *aeruginosa* (*i*.*e*. ≤50 µM; Fig. S1) were used throughout this study.

The transcriptional profiles of *P*. *aeruginosa* PAO1 grown to an A_600_ of 2.5 in LB in the presence or in the absence of 27 µM PAβN were compared by means of high-density oligonucleotide microarrays, by using Affimetrix GeneChip^®^ for *P*. *aeruginosa* PAO1. Following statistical validation of the dataset, only genes with a fold change >2 and a *p*-value <0.05 were considered for further analysis. Selected genes significantly up- or down-regulated by PAβN are listed in Tables 1 and 2, respectively (the complete gene list is given in Table S1, Supporting Information).

**Table 1:** Selected genes whose transcription is up-regulated by PAßN.

PA number^a^	Gene name^a^	Fold change^b^	Product name^a^
PA0509^*^	*nirN*	2.27	NirN
PA0510^*^	*nirE*	2.33	NirE
PA0511^*^	*nirJ*	2.23	heme*d*_1_ biosynthesis protein NirJ
PA0514^*^	*nirL*	2.3	heme*d*_1_ biosynthesis protein NirL
PA0516^*^	*nirF*	2.3	heme*d*_1_ biosynthesis protein NirF
PA0517^*^	*nirC*	3.53	probable *c*-type cytochrome precursor
PA0518^*^	*nirM*	3.32	cytochrome *c*_551_ precursor
PA0519^*^	*nirS*	4.48	nitrite reductase precursor
PA0523^*^	*norC*	2.87	nitric-oxide reductase subunit C
PA0524^*^	*norB*	5.51	nitric-oxide reductase subunit B
PA0525^*^	*norD*	2.19	probable denitrification protein NorD
PA1901^§^	*phzC1/C2*	2.24	phenazine biosynthesis protein PhzC
**PA1902** ^**§**^	*phzD1/D2*	2.26	phenazine biosynthesis protein PhzD
**PA1903** ^**§**^	*phzE1/E2*	2.22	phenazine biosynthesis protein PhzE
**PA1904** ^**§**^	*phzF1/F2*	2.11	probable phenazine biosynthesis protein
**PA2593**	*qteE*	2.06	quorum threshold expression element, QteE
PA3392^*^	*nosZ*	2.16	nitrous-oxide reductase precursor
PA4810^*^	*fdnI*	2.22	nitrate-inducible formate dehydrogenase, γ subunit

^a^PA number, gene name and product name are from the *Pseudomonas* Genome Database^23^. Genes previously reported as controlled by 3OC_12_-HSL are in bold characters^36–38^. ^*^Genes involved in nitrogen metabolism; ^§^Genes involved in phenazines biosynthesis.

^b^Fold change in gene expression in *P*. *aeruginosa* PAO1 grown in LB supplemented with 27 µM PAßN with respect to the same strain grown in LB.

**Table 2:** Selected genes whose transcription is down-regulated by PAßN.

PA number^a^	Gene name^a^	Fold change^b^	Product name^a^
PA0672^∫^	*hemO*	−4.81	hemeoxygenase
PA0676^∫^	*vreR*	−4.85	sigma factor regulator, VreR
PA0707	*toxR*	−2.12	transcriptional regulator ToxR
**PA1245**	*aprX*	−3.44	AprX
PA1912^∫^	*femI*	−2.35	ECF sigma factor, FemI
PA2385^∫^	*pvdQ*	−2.98	3OC_12_-homoserine lactone acylasePvdQ
PA2386^∫^	*pvdA*	−3.97	L-ornithine *N* ^5^-oxygenase
PA2394^∫^	*pvdN*	−2.85	PvdN
PA2395^∫^	*pvdO*	−2.32	PvdO
PA2396^∫^	*pvdF*	−3.22	pyoverdinesynthetase F
PA2397^∫^	*pvdE*	−3.16	pyoverdine biosynthesis protein PvdE
PA2398^∫^	*fpvA*	−2.03	ferripyoverdine receptor
PA2399^∫^	*pvdD*	−3.32	pyoverdinesynthetase D
PA2400^∫^	*pvdJ*	−3.36	PvdJ
PA2413^∫^	*pvdH*	−3.58	l-2,4-diaminobutyrate:2-ketoglutarate 4-aminotransferase
PA2424^∫^	*pvdL*	−3.73	PvdL
PA2425^∫^	*pvdG*	−2.58	PvdG
PA2426^∫^	*pvdS*	−10.48	sigma factor PvdS
**PA2570**	*lecA*	−2.42	LecA
PA3377^◊^	*phnJ*	−21.1	conserved hypothetical protein
PA3407^∫^	*hasAp*	−4.15	heme acquisition protein HasAp
PA3530^∫^	*bfd*	−2.54	bacterioferritin-associated ferredoxinBfd
PA4221^∫^	*fptA*	−2.52	Fe(III)-pyochelin outer membrane receptor precursor
PA4224^∫^	*pchG*	−2.05	pyochelin biosynthetic protein PchG
PA4225^∫^	*pchF*	−2.26	pyochelinsynthetase
PA4226^∫^	*pchE*	−2.06	dihydroaeruginoic acid synthetase
PA4228^∫^	*pchD*	−2.09	pyochelin biosynthesis protein PchD
PA4230^∫^	*pchB*	−2.69	salicylate biosynthesis protein PchB
PA4468	*sodM*	−11.65	superoxide dismutase
PA4470	*fumC1*	−8.96	fumaratehydratase
PA4708^∫^	*phuT*	−3.17	heme-transport protein, PhuT
PA4709^∫^	*phuS*	−3.37	PhuS
PA4710^∫^	*phuR*	−4.65	heme/hemoglobin uptake outer membrane receptor PhuR
PA5360^◊^	*phoB*	−15.28	two-component response regulator PhoB
PA5365^◊^	*phoU*	−9.4	phosphate uptake regulatory protein PhoU
PA5366^◊^	*pstB*	−14.02	ATP-binding component of ABC phosphate transporter
PA5367^◊^	*pstA*	−14.17	membrane protein component of ABC phosphate transporter
PA5369^◊^	*pstS*	−23.49	periplasmic phosphate-binding protein, PstS

^a^PA number, gene name and product name are from the *Pseudomonas* GenomeDatabase^23^. Genes previously reported as controlled by 3OC_12_-HSL are in bold characters^36–38^. ^∫^Genes previously reported to be controlled by iron starvation^25^; ^◊^Genes previously reported to be controlled by phosphate starvation^27^.

^b^Fold change in gene expression in *P*. *aeruginosa* PAO1 grown in LB supplemented with 27 µM PAßN with respect to the same strain grown in LB.

The transcription of 108 genes was significantly affected by PAβN (Table S1), corresponding to about 1.9% of *P*. *aeruginosa* PAO1 genes^23^. Of these, 39 genes were up-regulated and 69 genes were down-regulated in the presence of PAβN (Table S1). Among the 39 genes up-regulated by PAβN, the most represented categories comprise genes involved in nitrogen metabolism (*nir*, *nor* and *nos* genes; 33.3% of up-regulated genes) and in biosynthesis of phenazines (*phz* genes; 10.2% of up-regulated genes) (Tables 1 and S1). Phenazines constitute a group of nitrogen-containing heterocyclic compounds, including the virulence factor pyocyanin^24^.

Among the 69 down-regulated genes, 46 genes (66.7%) were previously reported to be repressed by iron^25^. These include almost all the genes involved in the biosynthesis, uptake and regulatory response to the siderophores pyoverdine and pyochelin, including the *pvdS* sigma factor gene, which also activates the expression of *prpL* protease and *toxA* toxin genes (Tables 2 and S1). Moreover, metabolic and virulence genes previously shown to be induced in response to iron starvation were down-regulated by PAβN, including fumarate hydratase (*fumC*1), superoxide dismutase (*sodM*) and protease (*aprX*) genes (Table 2). The negative effect exerted by PAβN on the iron-starvation response pathway correlates with previous studies showing that PAβN synergizes with iron chelators in reducing the growth rate and biofilm formation of *P*. *aeruginosa* ^26^. Moreover, PAβN treatment caused down-regulation of genes repressed by phosphate availability, including *pho*, *pst* and *pnh* genes^27^ (Tables 2 and S1). Overall, the expression of many genes important for *P*. *aeruginosa* pathogenicity, such as *pvdS*, *phoB*, *pstS* and *vreR* ^25–30^, was strongly repressed by PAβN (Table 2).

**Figure 1:**
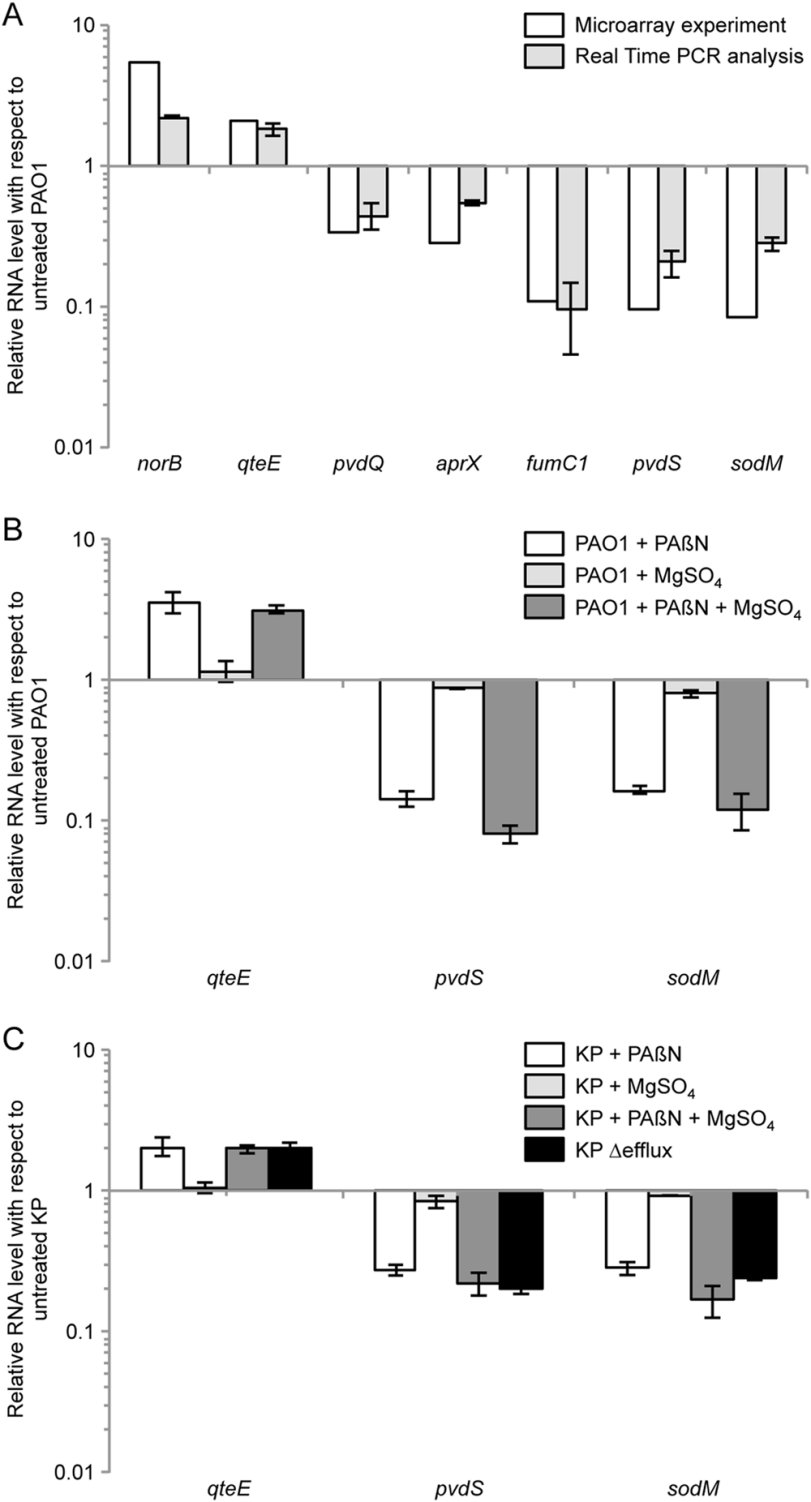
Validation of the microarray data by qRT-PCR. mRNA levels of the indicated genes quantified by qRT-PCR in: (**A**) The *P*. *aeruginosa* PAO1 strain grown to an A_600_ of 2.5 in LB supplemented with 27 µM PAßN, relative to the same strain grown in LB (grey bars), in comparison with microarray data for the same genes (white bars); (**B**) The *P*. *aeruginosa* PAO1 strain grown to an A_600_ of 2.5 in LB supplemented with 27 µM PAßN (white bars), with 1 mM MgSO_4_ (light-grey bars), or with 27 µM PAßN plus 1 mM MgSO_4_ (dark-grey bars) relative to the same strain grown in LB; (**C**) The *P*. *aeruginosa* PAO1-KP strain grown to an A_600_ of 2.5 in LB supplemented with 27 µM PAßN (white bars), with 1 mM MgSO_4_ (light-grey bars), or with 27 µM PAßN plus 1 mM MgSO_4_ (dark-grey bars), and the *P*. *aeruginosa* PAO1-KP ∆efflux strain grown to an A_600_ of 2.5 in LB (black bars), relative to the PAO-KP strain grown in LB. The average of two independent analyses performed on three technical replicates is shown with SD.

The differential expression of selected genes identified as PAβN-controlled was validated by quantitative reverse transcription PCR (qRT-PCR) analysis performed on *P*. *aeruginosa* cultures grown under the same conditions as those used for the microarray experiment. The qRT-PCR results matched the microarray data, since the mRNA level of the *norB* and *qteE* genes increased in the presence of 27 µM PAβN, while the mRNA level of the *pvdQ*, *aprX*, *fumC*1, *pvdS* and *sodM* genes decreased in the same conditions (Fig. 1A).

Despite the concentration of PAβN used in this experiment (27 µM) is not expected to destabilize the cell membrane of wild type PAO1, the possibility that this EPI controls some of the identified genes *via* membrane perturbation rather than efflux pump inhibition cannot be ruled out. However, the specificity of PAβN effect as an EPI in our settings is supported by the observation that only 2 out of the 108 PAβN-regulated genes (*i*.*e*. *phzF1* and PA4139; Table S1) were identified in a previous microarray analysis performed with sub-MIC concentration of the membrane destabilizing peptide polymyxin E (colistin)^31^ (Table S1). Furthermore, none of the genes whose expression was altered upon exposure to sub-MIC concentration of polymyxin B^32^ were affected by PAβN.

Additional qRT-PCR analyses were also performed to further support the primary role of PAβN as an EPI. Since previous reports showed that 1 mM Mg^2+^ completely abolished the permeabilizing effect exerted by PAβN on bacterial membranes^19, 33^, the effect of PAβN on the mRNA level of *qteE*, *pvdS* and *sodM* was compared in the absence and in the presence of 1 mM MgSO_4_. The expression of the same genes was also evaluated in a *P*. *aeruginosa* efflux-deficient mutant (PAO1-KP Δefflux) carrying deletions in genes encoding the four major RND efflux pumps of this bacterium, namely MexAB-OprM, MexCD-OprJ, MexEF-OprN and MexXY-OprM^34^ (Table S2). Since this mutant was not generated in our laboratory, and it is well known that PAO1 strains maintained in different laboratories disclose genotype variability^35^, strain PAO1-KP Δefflux was compared with its isogenic wild type strain PAO1-KP^34^.

This experiment revealed that 27 µM PAβN increases the mRNA level of *qteE* and decreases the mRNA level of *pvdS* and *sodM* irrespective of the presence or the absence of MgSO_4_, both in PAO1 (Fig. 1B) and in PAO1-KP (Fig. 1C). Notably, the fold change in the mRNA level of the tested genes was similar in PAO1-KP supplemented with PAβN and in PAO1-KP Δefflux relative to untreated PAO1-KP (Fig. 1C), supporting the conclusion that the alteration in gene expression caused by PAβN relies on its ability to inhibit efflux pumps, rather than on its membrane permeabilizing effect. This is in line with previous reports suggesting that PAβN has a strong activity as an efflux pump inhibitor and a weak, concentration-dependent activity in destabilizing the cell envelope, both in *P*. *aeruginosa* and in *Escherichia coli* ^19, 33^. Unfortunately, the well-known toxic effect of PAβN to efflux pumps-deficient *P*. *aeruginosa* cells^2, 19^ does not allow to investigate the effect of PAβN on the PAO1-KP Δefflux strain.

Overall, these data indicate that the PAβN-dependent inhibition of efflux pumps has a profound impact on the *P*. *aeruginosa* transcriptome.

### PAβN treatment affects *P*. *aeruginosa* virulence-related phenotypes

**Figure 2:**
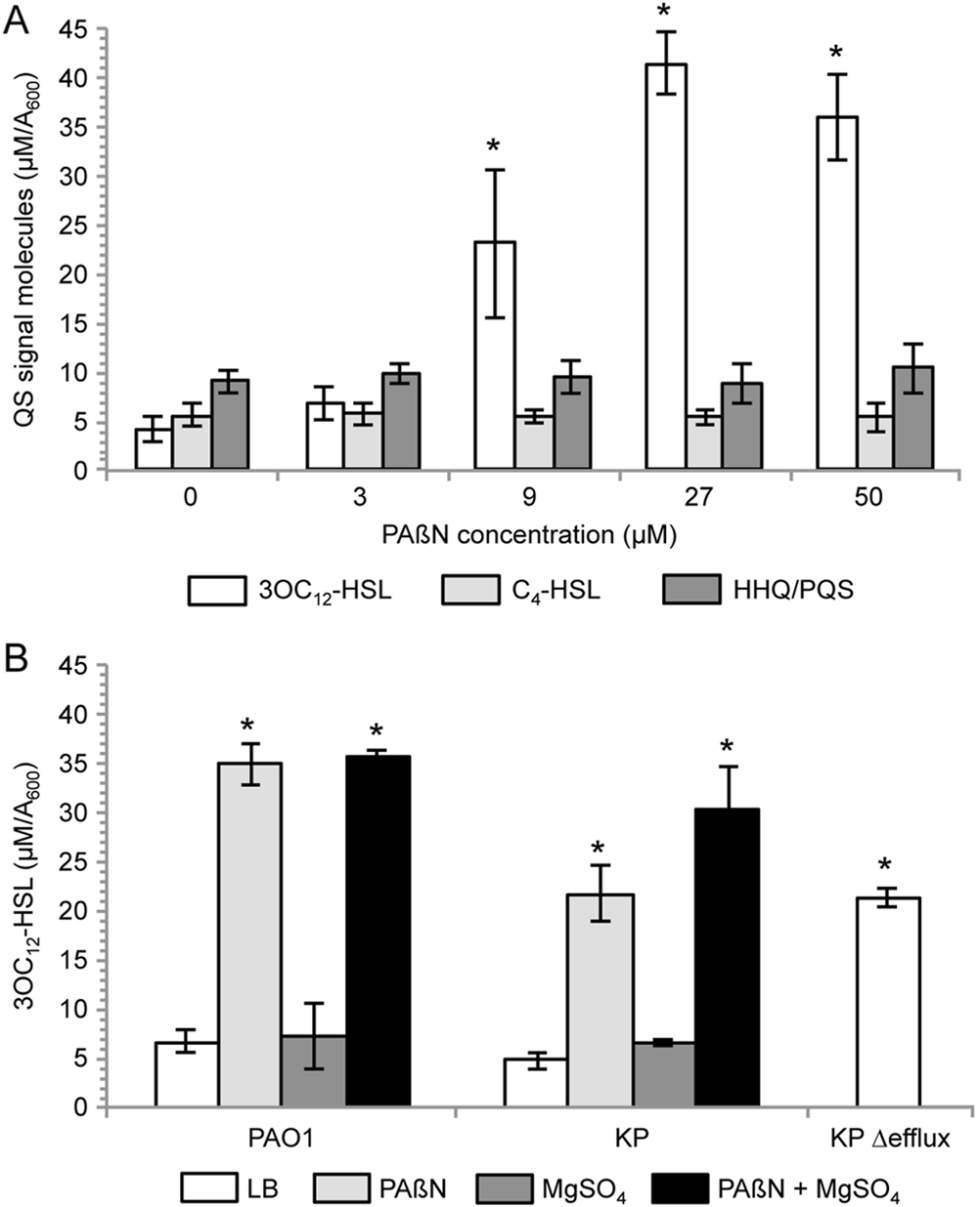
Effect of PAβN on QS signal molecules production. (**A**) 3OC_12_-HSL (white bars), C_4_-HSL (light-grey bars) and HHQ/PQS (dark-grey bars) production in *P*. *aeruginosa* PAO1 stationary phase cultures grown in LB or in LB supplemented with PAßN at the concentrations indicated below the histogram. (**B**) 3OC_12_-HSL production in the indicated strains grown in LB (white bars), or in LB supplemented with 27 µM with PAßN (light-grey bars), with 1 mM MgSO_4_ (dark-grey bars), or with 27 µM PAßN plus 1 mM MgSO_4_ (black bars). The average of at least three independent experiments is reported with SD; statistical significance with respect to the untreated sample is indicated with one asterisk (*p* < 0.05).

The expression of the genes involved in 3OC_12_-HSL and C_4_-HSL synthesis and reception (*i*.*e*. *lasI*-*lasR*, and *rhlI*-*rhlR*, respectively) and of the vast majority of genes known to be controlled by these QS signal molecules^36–38^ was not inhibited by PAβN in the microarray analysis (Table S1). This result and the positive effect exerted by PAβN on the expression of pyocyanin biosynthetic genes was not expected, since PAβN was previously shown to negatively affect the transcription of the *las* and *rhl* QS genes and the expression of phenotypes controlled by QS (*i*.*e*. pyocyanin, proteases and elastase production) in *P*. *aeruginosa* strains isolated from urinary tract and wound infections^21^. To clarify this issue, we measured the level of QS signals (*i*.*e*. 3OC_12_-HSL, C_4_-HSL and HHQ/PQS) and of the above-mentioned QS-dependent virulence factors in supernatants collected from *P*. *aeruginosa* PAO1 cultures in LB supplemented with increasing concentrations of PAβN (experimental details are given in Materials and Methods). Results showed that the production of 3OC_12_-HSL is significantly increased in the presence of PAβN concentrations ≥9 µM (Fig. 2A), while C_4_-HSL and HHQ/PQS production was not affected even at the maximum PAβN concentration tested (50 µM; Fig. 2A). The observation that PAβN increases 3OC_12_-HSL production both in PAO1 and in PAO1-KP also in the presence of 1 mM MgSO_4_, and that 3OC_12_-HSL levels are higher in the supernatant of PAO1-KP Δefflux relative to the supernatant of PAO1-KP (Fig. 2B) indicates that the effect of PAβN on 3OC_12_-HSL can be ascribed to the inhibition of efflux pumps, rather than to membrane perturbation. Further experiments carried out with transcriptional fusions confirmed that PAβΝ did not affect *lasI* and *lasR* promoter activity in PAO1 (Fig. S2), in agreement with the microarray data. Hence, the positive effect exerted by PAβΝ on 3OC_12_-HSL production in *P*. *aeruginosa* does not appear to occur at the transcriptional level. Interestingly, PAβΝ reduced the transcription of *pvdQ* (Fig. 1 and Table 2), a gene coding for the PvdQ acylase, an enzyme responsible for 3OC_12_-HSL degradation^39^. Therefore, the increase in 3OC_12_-HSL level caused by PAβΝ could be due, at least in part, to a decreased degradation of this signal molecule as a consequence of *pvdQ* down-regulation. In addition, PAβΝ enhanced the transcription of *qteE* (Fig. 1 and Table 1), a gene coding for a protein that hampers the activity of the 3OC_12_-HSL-receptor protein LasR^40^. The enhanced expression of QteE in PAβΝ-treated cells may result in reduced levels of active LasR, thus counterbalancing the effect of increased 3OC_12_-HSL levels on the transcription of LasR-dependent genes.

**Figure 3:**
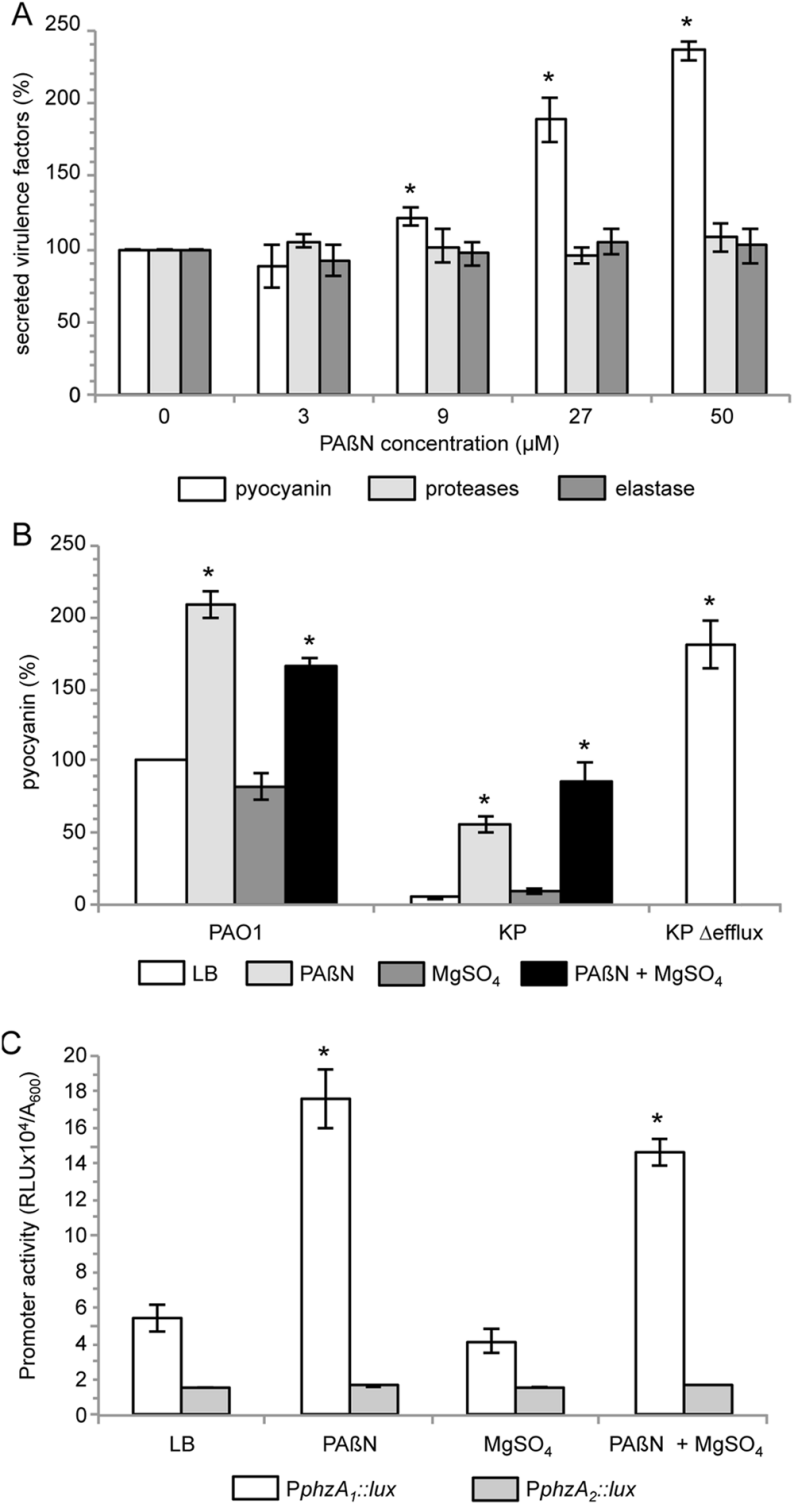
Effect of PAβN on pyocyanin production. (**A**) Pyocyanin (white bars), proteases (light-grey bars) and elastase (dark-grey bars) production in *P*. *aeruginosa* PAO1 cultures grown in LB in the absence or in the presence of PAßN at the concentrations indicated below the histogram. (**B**) Pyocyanin production in the indicated strains grown in LB (white bars), or in LB supplemented with 27 µM PAßN (light-grey bars), with 1 mM MgSO_4_ (dark-grey bars), or with 27 µM PAßN plus 1 mM MgSO_4_ (black bars). Pyocyanin production of strain PAO1 grown in LB is considered as 100%. (**C**) P*phzA*_1_ (white bars) and P*phzA*_2_ (grey bars) promoter activity measured in *P*. *aeruginosa* PAO1 cultures grown in LB or in LB supplemented with 27 µM PAßN, with 1 mM MgSO_4_, or with 27 µM PAßN plus 1 mM MgSO_4_, as indicated below the histogram. The average of at least three independent experiments is reported with SD; statistical significance with respect to the untreated sample is indicated with one asterisk (*p* < 0.05).

As shown in Fig. 3A, pyocyanin production increased in the presence of PAβN concentrations ≥9 µM by comparison with the untreated control. Conversely, PAβN did not affect the production of proteases and elastase (Fig. 3A). These results are in agreement with the microarray data, showing that PAβN increases the transcription of pyocyanin biosynthetic genes in PAO1, without affecting the mRNA level of proteases and elastase genes (Tables 1 and S1). Therefore, it can be argued that the positive effect exerted by PAβN on pyocyanin production in *P*. *aeruginosa* PAO1 is likely exerted *via* a QS-independent pathways controlling phenazines biosynthesis. The increase in pyocyanin levels caused by PAβN treatment was maintained in the presence of MgSO_4_ in both PAO1 and PAO1-KP, although the absolute pyocyanin levels were lower in PAO1-KP than in PAO1 (Fig. 3B). Moreover, pyocyanin production in PAO1-KP Δefflux was significantly increased relative to PAO1-KP (Fig. 3B). These observations suggest that pyocyanin production is affected by PAβN *via* specific EPI activity. High sequence homology of pyocyanin biosynthetic operons *phzA*_*1*_-*G*_*1*_ and *phzA*_2_ *-G*_2_ in PAO1^23^ does not allow discriminating their mRNAs *via* microarray or qRT-PCR analyses. Therefore, transcriptional fusions between the P*phzA*_1_ or P*phzA*_2_ promoters and the *luxCDABE* operon^41^ were used to clarify the effect of PAβN on the pyocyanin biosynthetic operons. As shown in Fig. 3C, PAβN increased the activity of the P*phzA*_1_ promoter, while it did not affect P*phzA*_2_. Also in this case, the effect of PAβN was not alleviated in the presence of MgSO_4_ (Fig. 3C).

**Figure 4:**
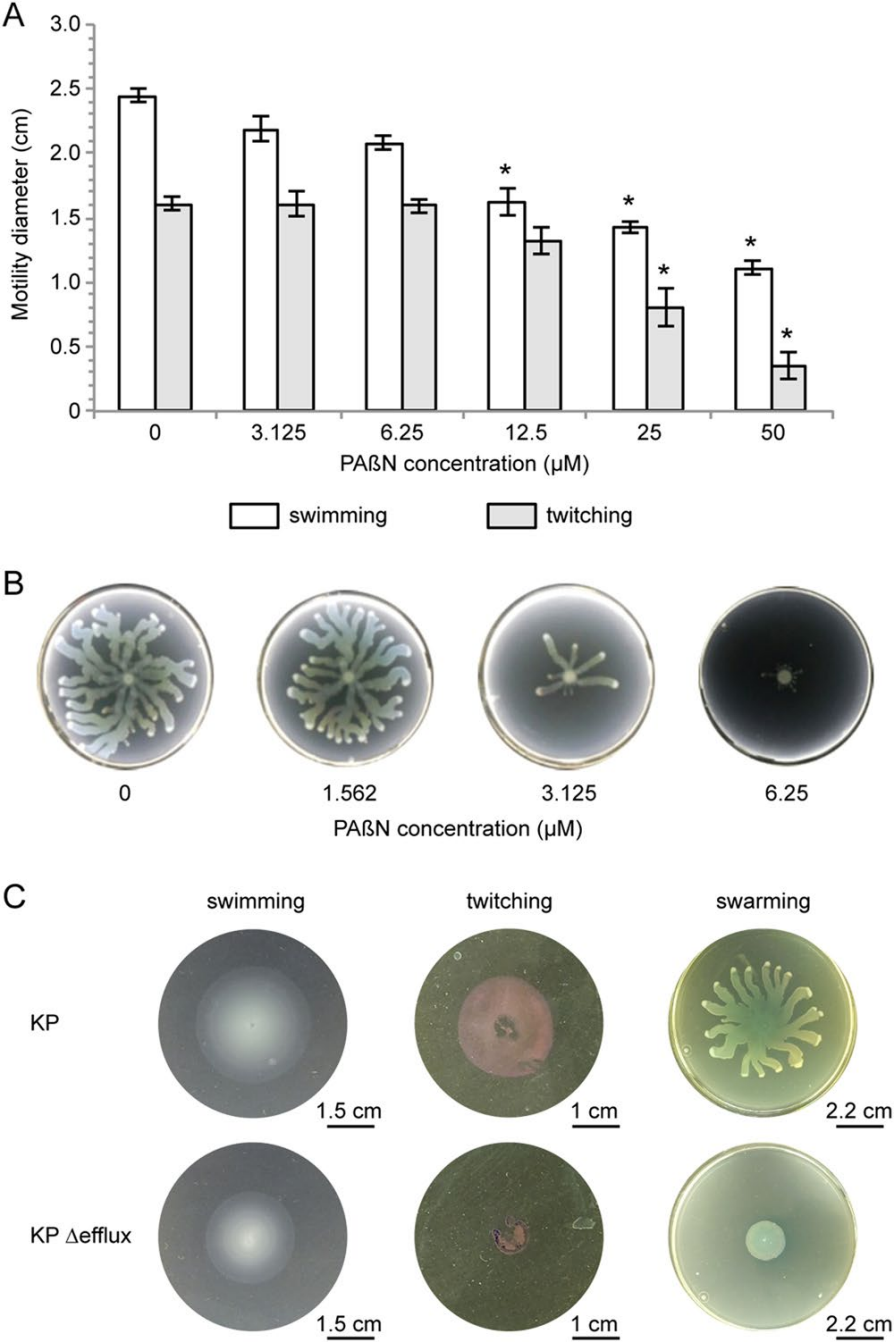
Effect of PAβN on *P*. *aeruginosa* motility. (**A**) *P*. *aeruginosa* swimming (white bars) and twitching (grey bars) motility in the absence or in the presence of PAßN at the concentrations indicated below the histogram. The average diameter of five independent experiments is reported with SD; statistical significance with respect to the untreated control sample is indicated with one asterisk (*p* < 0.05). (**B**) Images of *P*. *aeruginosa* swarming plates supplemented with the indicated concentrations of PAßN. (**C**) Images of *P*. *aeruginosa* PAO1-KP and PAO1-KP ∆efflux swimming, twitching and swarming plates. For the swarming assay, images of the entire plates are reported, while for swimming and twitching assays, magnification of the halos are shown. Twitching halos were stained with crystal violet. One representative experiment out of three independent replicates is shown for (**B**) and (**C**).

Additional phenotypic analyses revealed that 50 µM PAβN caused a 8-fold and 2-fold reduction of twitching and swimming motility compared with the untreated control, respectively (Fig. 4A). Moreover, swarming motility was completely abrogated in the presence of 6.25 µM PAβN (Fig. 4B), in agreement with previous observations on *P*. *aeruginosa* clinical isolates^21^. A substantial decrease in swimming, twitching and swarming was also observed in PAO1-KP Δefflux relative to PAO1-KP (Fig. 4C), indicating that the effect of PAβΝ on these phenotypes is mainly dependent on efflux pumps inhibition. The negative effect exerted by PAβΝ on *P*. *aeruginosa* motility seems to be unrelated to an altered expression of pili, flagella or rhamnolipids biosynthetic genes, as suggested by the microarray results (Table S1). However, motility is a pleiotropic and energetically demanding process, strongly affected by nutrients availability. In this context, the metabolic alteration caused by PAβΝ (*e*.*g*. up-regulation of nitrogen metabolism genes and down-regulation of iron-uptake genes; Tables 1 and S1) could explain the effect of this EPI on *P*. *aeruginosa* motility. Moreover, it is well documented that *pvdQ* is up-regulated in swarming cells, while its deletion abrogates swarming motility in *P*. *aeruginosa* ^42^. Thus the PAβΝ-mediated reduction of *pvdQ* transcription (Fig. 1 and Table 1) correlates with the strong inhibitory effect exerted by this EPI on swarming motility.

In summary, the effects of PAβΝ on *P*. *aeruginosa* PAO1 QS and virulence-related phenotypes are in agreement with the microarray analysis, and confirm that this molecule increases 3OC_12_-HSL and pyocyanin levels *via* specific EPI activity, without affecting the production of other QS signal molecules and of the QS-controlled virulence factors elastase and proteases.

### *In vivo* anti-virulence activity of PAβN

The above results show that PAβN (≤50 µM) inhibits *P*. *aeruginosa* PAO1 processes related to motility and acquisition of micronutrients (*i*.*e*. phosphate and iron), relevant for pathogenesis in several models of acute infection^25, 27–30^. On the other hand, in the PAO1 strain PAβN stimulates the production of both 3OC_12_-HSL and pyocyanin, both playing a positive role in *P*. *aeruginosa* virulence^10, 28, 43–45^.

**Figure 5:**
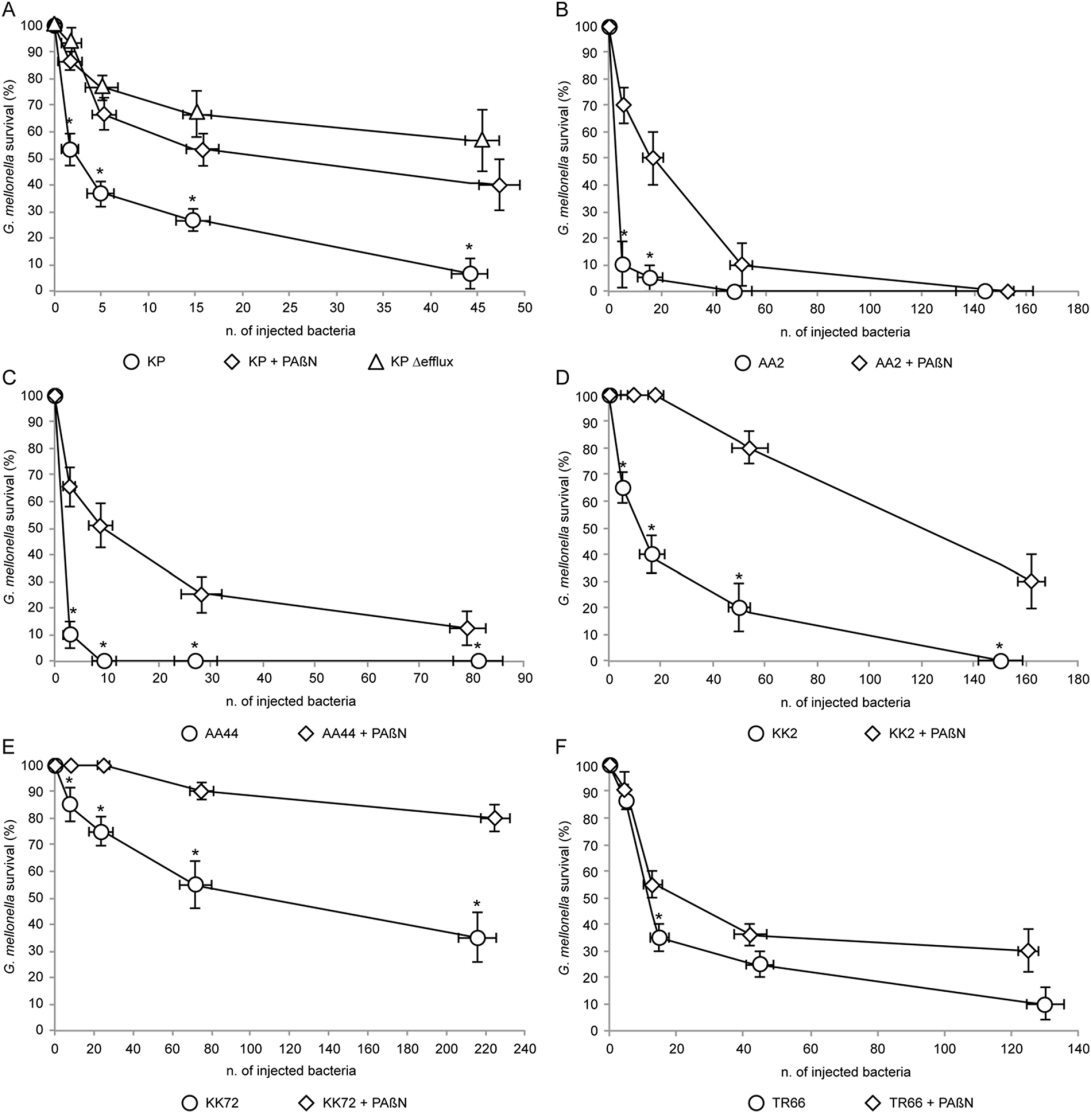
Effect of PAβN on *P*. *aeruginosa* virulence in *G*. *mellonella* larvae. Viability of *G*. *mellonella* larvae 24 h after injection with the indicated amount of bacteria. Larvae were injected with: (**A**) The *P*. *aeruginosa* strains PAO-KP wild type (circles), PAO-KP wild type in the presence of PAßN at ca. 50 µM final concentration (diamonds), or PAO1-KP Δefflux (triangles); (**B**–**F**) The indicated *P*. *aeruginosa* CF isolates in the absence (circles) or in the presence of PAßN at ca. 50 µM final concentration (diamonds). Mean values from three independent experiments, each performed on at least 30 larvae, are reported with SD; statistical significance with respect to the larvae challenged with the indicated strains in the absence of PAßN is indicated with one asterisk (*p* < 0.05).

These puzzling *in vitro* results raise the question of what kind of effect PAβN has on *P*. *aeruginosa* PAO1 virulence *in vivo*. To tackle this issue, the effect of PAβN on *P*. *aeruginosa* virulence was assessed in *Galleria mellonella*, an insect model of infection that well correlates with murine acute infection models^28^. We firstly aimed at validating the infection model by testing the virulence of the efflux-deficient mutant PAO1-KP Δefflux compared to its isogenic wild type strain PAO1-KP. The survival rate of *G*. *mellonella* larvae 24 h after the challenge with the tested *P*. *aeruginosa* strains is shown in Fig. 5A. Nearly all larvae infected with wild type *P*. *aeruginosa* (PAO1-KP) were killed at the maximum infective dose tested (ca. 45 colony forming units or CFU/larva), and larvae survival increased as a function of decreasing infective dose. Conversely, >50% of larvae challenged with the efflux-deficient mutant PAO1-KP Δefflux survived also at the maximum infective dose tested. The differences between the wild type and the efflux-deficient mutant survival curves were evident at all infection doses (Fig. 5A). To the best of our knowledge, this result is the first demonstration that genetic inactivation of RND efflux pumps causes a decrease in *P*. *aeruginosa* pathogenic potential *in vivo*. Interestingly, a PAO1 triple mutant inactivated in MexAB-OprM, MexCD-OprJ and MexEF-OprN did not show reduced virulence in the same infection model in a previous study^46^. This observation suggests that the deletion of MexXY-OprM in addition to MexAB-OprM, MexCD-OprJ and MexEF-OprN in PAO1-KP Δefflux could be critical to reduce the virulence potential of *P*. *aeruginosa* in the *G*. *mellonella* model of infection. However, this issue should be investigated by using identical experimental settings and isogenic mutants generated in the same PAO1 strain.

Since the average weight of *G*. *mellonella* larvae was ca. 500 mg, and arbitrarily assuming uniform dispersal of injected bacteria and PAβN in 500 µl of internal volume in each larva^43^, we calculated that to reach 50 µM final concentration of PAβN, each larva should be injected with 25 µl of saline containing 1 mM PAβN. As a preliminary control experiment, we verified that the injection of 25 µl of saline containing 1 mM PAβN did not affect the survival of uninfected larvae (data not shown). Then, *G*. *mellonella* larvae were inoculated with *P*. *aeruginosa* PAO1-KP in the absence or in the presence of PAβN. Results shown in Fig. 5A demonstrate that PAβN was able to protect *G*. *mellonella* larvae from *P*. *aeruginosa* PAO1-KP infection. Interestingly, the survival plot of the larvae infected with PAO1-KP and treated with PAβN was slightly lower than that of the untreated larvae infected with the efflux-deficient mutant PAO1-KP ∆efflux, but was higher than the untreated control infected with wild type PAO1-KP, supporting the hypothesis that PAβN-mediated inhibition of RND efflux pumps is the cause of virulence attenuation. Also in this case, it was not possible to directly verify this hypothesis by testing the effect of PAβN on PAO1-KP ∆efflux infectivity in *G*. *mellonella* due to the toxicity exerted by PAβN on this mutant strain^19–22^. Notably, PAβN exerted a similar protective effect when the larvae were challenged with the *P*. *aeruginosa* PAO1 strain routinely used in our laboratory (data not shown). Overall, these results strongly suggest that the PAβN-mediated inhibition of RND efflux pumps decreases *P*. *aeruginosa* PAO1 pathogenicity in *G*. *mellonella*, despite the increase in 3OC_12_-HSL and pyocyanin levels observed *in vitro* in response to PAβN.

### Effect of PAβN on *P*. *aeruginosa* cystic fibrosis isolates

The previous observation that PAβN treatment inhibited 3OC_12_-HSL and pyocyanin production in *P*. *aeruginosa* clinical strains^21^ and our results showing that this EPI has an opposite effect in the reference laboratory strains PAO1 and PAO1-KP suggest that virulence-related phenotypes could be variably affected by PAβN, depending on the test strain.

In order to verify this hypothesis, we measured the effect of 27 µM PAβN treatment on 3OC_12_-HSL and pyocyanin production, as well as swarming motility, in eleven *P*. *aeruginosa* clinical strains isolated from the lungs of cystic fibrosis (CF) patients^47^. The growth curve of all tested strains was not affected by PAβN treatment (data not shown). Interestingly, among the seven isolates producing 3OC_12_-HSL, PAβN increased this phenotype in one strain (*i*.*e*. KK72), had no effect in two strains (*i*.*e*. AA2 and KK71), while inhibited the production of this signal molecule in the remaining strains, though to different extents (Table 3). Out of four CF isolates that produced detectable amounts of pyocyanin, two responded to PAβN by reducing and two by increasing pyocyanin production (Table 3). Finally, only five CF isolates showed swarming motility in the absence of PAβN, and this phenotype was abrogated upon PAβN treatment in all of them (Table 3).

**Table 3:** Effects of PAßN treatment on virulence phenotypes in *P*. *aeruginosa* clinical isolates.

Strain^a^	3OC_12_-HSL^b^	Pyocyanin^b^	Swarming motility^b^
AA2	102%	86%	<10%
AA11	NP	125%	NS
AA12	24%	73%	NS
AA43	NP	NP	<10%
AA44	59%	142%	<10%
KK2	74%	NP	NS
KK27	81%	NP	NS
KK71	97%	NP	NS
KK72	134%	NP	NS
TR1	NP	NP	<10%
TR66	NP	NP	<10%

^a^ *P*. *aeruginosa* strains isolated from cystic fibrosis patients^47^.

^b^Percentage of 3OC_12_-HSL levels, pyocyanin production or swarming motility in the presence of 27 µM PAßN with respect to the untreated control. The average of at least three independent experiments is reported; SD ≤ 10%. NP, non producer strain; NS, non swarmer strain.

It appears therefore that PAβN has variable effects on QS signal and pyocyanin production, which are strain-dependent. This is in agreement with the previous study^21^, showing that PAβN inhibited to a different extent 3OC_12_-HSL and C_4_-HSL levels in two isolates from urinary tract infections, while C_4_-HSL production was not inhibited in two isolates from wound infections. The extent of PAβN-mediated inhibition on all the tested virulence-related phenotypes varied significantly among the four clinical isolates previously analysed^21^. In contrast, swarming motility was invariably inhibited in all swarming-proficient CF isolates (Table 3). Moreover, for the majority of isolates, no correlation was observed between production/inhibition of the 3OC_12_-HSL signal molecule and the effect of PAβN on pyocyanin levels or swarming motility (Table 3), supporting our hypothesis that the effect of PAβN on pyocyanin production is exerted *via* QS-independent pathway(s).

The anti-virulence activity of PAβN against CF clinical isolates was further investigated in *G*. *mellonella* larvae in the presence and in the absence of 50 µM PAβN. The CF isolates AA2, AA44, KK2, KK72, and TR1 were selected based on their different pattern of virulence phenotypes and sensitivity to PAβN (Table 3). Since the CF isolates showed different pathogenicity in the *G*. *mellonella* larvae, the optimal range of injected bacteria to be used in the infection was preliminarily assessed (data not shown). As shown in Fig. 5C, D and E, PAβN significantly increased the survival of *G*. *mellonella* larvae challenged with the strains AA44, KK2 and KK72 at all the tested infective doses. Conversely, protection from AA2 infection was observed only for low doses of injected bacteria (<20 bacteria per larva; Fig. 5B), and poor protection effect was observed when *G*. *mellonella* larvae where challenged with the TR66 isolate (Fig. 5F).

Overall, PAβN exerted a general protective effect on *G*. *mellonella* larvae against *P*. *aeruginosa* CF isolates (Fig. 5B–F), irrespective of its positive or negative influence on 3OC_12_-HSL and pyocyanin production (Table S3).

## Conclusions

Efflux pumps inhibition is a viable strategy to overcome the problem of antibiotic resistance. Both academic and industrial research is currently directed to the development of efflux inhibitors, and the interest in RND efflux pump inhibitors as antibiotic adjuvants is steadily increasing over the years^2, 5–7^. Moreover, the notion that RND efflux pumps could play a role in bacterial infection is emerging^8, 9^, implying that certain EPIs could also be endowed with anti-virulence properties. Nevertheless, EPIs are usually considered only for their properties as antibiotic adjuvants, while their anti-virulence potential is seldom taken into account.

Here we demonstrate in a simple infection model that RND efflux pumps contribute to the establishment of *P*. *aeruginosa* PAO1 infection and, accordingly, that the EPI PAβN is able to reduce pathogenicity. In PAO1, the protective effect exerted by PAβN *in vivo* well correlates with *in vitro* suppression of some virulence-related phenotypes and repression of key virulence-related genes.

Although this study was not aimed at investigating the mechanistic link between RND efflux pumps and virulence, our findings provide relevant hints for future research. The transcriptomic analysis showed that the effect of PAβN on *P*. *aeruginosa* PAO1 physiology is specific, since it affects particular groups of genes, mainly related to iron and phosphate acquisition, as well as nitrogen metabolism. It is particularly relevant that PAβN inhibits the transcription of global regulators that are crucial for the establishment of a productive infection, such as the sigma factor gene *pvdS* and the response regulator gene *phoB*, controlling the regulons responding to iron and phosphate starvation, respectively^48, 49^.

It should be noticed that PAβN may also destabilize the outer membrane of Gram-negative bacteria, in addition to act as a nonspecific RND efflux pump inhibitor^19, 22, 33^. However, the majority of studies agree that the membrane-destabilizing activity of this molecule is only relevant in strains unable to extrude PAβN (*i*.*e*. mutants lacking RND efflux pumps), and that PAβN mainly acts as an efflux inhibitor in efflux pump-proficient isolates^19, 33^. Here, PAβN had no effect on the growth rate of *P*. *aeruginosa* at concentrations up to 50 µM (Fig. S1), showing that in our experimental setting PAβN does not have growth-limiting effects. Most of the transcriptional and phenotypic effects observed in this study are controlled by PAβN also in the presence of the membrane stabilizing ion Mg^2+^, and are mimicked by deletion of multiple efflux pumps in the PAO1-KP ∆efflux mutant, strongly suggesting that, in our experimental setting, PAβN mainly acts as an efflux pump inhibitor.

By combining the response of clinical *P*. *aeruginosa* isolates to PAβN *in vitro* (Table 3) and *in vivo* (Fig. 5), no correlation could be established between the effect of this EPI on some virulence phenotypes (*i*.*e*. 3OC_12_-HSL and pyocyanin production, swarming motility) and the outcome of *G*. *mellonella* infection. This evidence suggests either that the protective effect of PAβN *in vivo* occurs through inhibition of virulence-related trait(s) not investigated in this study, or that the specific virulence factors affected by PAβN may be strain-specific.

Although the number of strains and virulence-related phenotypes tested here and in the previous study^21^ is not sufficient to drive a definitive conclusion, the strain-dependent response to PAβN is an issue that deserves to be taken into consideration when testing the anti-virulence properties of any EPI. Unfortunately, PAβN is toxic for humans, hindering future therapeutic application and discouraging further studies aimed at characterizing the effect of this specific EPI on a wider panel of *P*. *aeruginosa* clinical strains. Actually, toxicity toward human cells is one of the major obstacle for microbial EPI implementation, and more efforts directed at specifically inhibiting efflux pumps operating only in prokaryotes are required. However, the search for new EPI candidates with improved pharmacological properties with respect to PAβN is in progress, as testified by the many research articles and thoughtful reviews published on this topic^2, 5–7^.

In conclusion, this study shows that RND efflux pump inhibition has an impact on bacterial virulence *in vivo*, and highlights that any new EPI should be tested not only for its ability to increase the inhibitory activity of antibiotics, but also for its anti-virulence effect. Given the strain-dependent response of *P*. *aeruginosa* to PAβN, anti-virulence properties should be tested on different virulence traits and on large panels of *P*. *aeruginosa* isolates from different types of infection.

## Materials and Methods

### Bacterial strains, growth conditions and chemicals

*P*. *aeruginosa* strains used in this study are listed in Table S2. All strains were routinely grown in Lysogeny Broth (LB)^50^ supplemented with 50 mM 3-(*N*-morpholino)propanesulfonic acid (MOPS), pH 7.0. PAβN (Sigma-Aldrich) was suspended in dimethyl sulfoxide (DMSO) at a 10 mM final concentration.

### Measurements of promoter activity and phenotypic assays

*P*. *aeruginosa* PAO1 strains carrying the P*phzA*_1_ *::luxCDABE* or P*phzA*_2_ *::luxCDABE* transcriptional fusions^41^ were grown at 37 °C for 10 h in LB or in LB supplemented with 27 µM PAβN, 1 mM MgSO_4_ or 27 µM PAβN plus 1 mM MgSO_4_. Bioluminescence was determined in the resulting cultures as a function of cell density using an automated luminometer-spectrometer (Tecan Spark 10M), as previously described^41^.

Levels of QS signal molecules in *P*. *aeruginosa* PAO1, PAO1-KP and PAO1-KP ∆efflux culture supernatants were determined during bacterial growth in LB supplemented with different PAβN concentrations and/or 1 mM MgSO_4_, by using the reporter strains specific for 3OC_12_-HSL, C_4_-HSL and HHQ/PQS^43, 51, 52^.

Pyocyanin was extracted with 3 ml of chloroform from 5 ml of cell-free supernatants of *P*. *aeruginosa* PAO1, PAO1-KP and PAO1-KP ∆efflux cultures grown at 37 °C for 10 h in LB supplemented with different PAβN concentrations and/or 1 mM MgSO_4_, and then re-extracted into 1 ml of 0.2 N HCl. The A_520_ of the resulting solution was measured to determine pyocyanin level^43, 44^. Proteases and elastase activities were determined in 100 µl of the same cell-free supernatants by the azocasein and elastin-Congo red hydrolysis assays, respectively^43, 44^.

Swimming, swarming and twitching motilities were assessed as previously described^43, 44^.

### Transcriptomic analysis

*P*. *aeruginosa* PAOI was inoculated at an A_600_ of 0.01 into 20 ml of LB with or without 27 μM PAβN. The cultures were grown at 37 °C with shaking until they reached an A_600_ of 2.5, and then 1 ml of cells was harvested by centrifugation. RNA extraction, retro-transcription and high-density oligonucleotides microarrays transcriptome analysis were performed and analysed as previously described^44, 52^. RNA integrity was monitored by agarose gel electrophoresis, and the absence of contaminating chromosomal DNA was verified by PCR with primers pairs FW*pqsB*-RV*pqsB* and FW16SRT-RV16SRT (Table S3).

Processing of the *P*. *aeruginosa* PAO1 Affimetrix GeneChip^®^ and statistical analysis of the dataset were performed at Lausanne Genomic Technologies Facility, Center for Integrative Genomics, University of Lausanne, Switzerland. For each condition, two different pools of RNA were compared (biological duplicate), each containing RNAs from three independent extractions (technical triplicate). Fold changes >2.0 with a *p*-value < 0.05 were considered as statistically significant.

### qRT-PCR analyses

Novel *P*. *aeruginosa* PAO1 cultures were prepared specifically for qRT-PCR analysis. Growth conditions, and sampling for RNA extraction were the same used for the microarray experiments described above. When required, LB was also supplemented with 1 mM MgSO_4_. The same setting were used also for qRT-PCR analysis performed in PAO-KP and PAO1-KP ∆efflux. cDNA synthesis was performed from 1 µg of total purified RNA by using random hexamer primers and the iScript Reverse Transcription Supermix for RT-qPCR kit (BioRad).

qRT-PCR reactions were performed using the iTaq™ Universal SYBR^®^ Green Supermix (BioRad) and primers listed in Table S3, which were designed using the Primer-Blast software (www.ncbi.nlm.nih.gov/tools/primer-blast). The reaction involved incubation at 95 °C for 1 min and 40 cycles of amplification at 95 °C for 10 s and 60 °C for 45 s. The 16 S ribosomal RNA was used as the internal control to calculate the relative fold change in gene expression by the 2^−∆∆Ct^ method^53^. The analysis was performed in duplicate on three technical replicates.

### *Galleria mellonella* killing assay

The *G*. *mellonella* killing assay was performed as previously described^43^, with minor modifications. Briefly, *G*. *mellonella* caterpillars in the final instar larval stage (average weight, 480 ± 70 mg) were infected with 25 µl of bacterial cell suspensions in saline containing or not 1 mM PAβN. Although *P*. *aeruginosa* cells were incubated in the presence of PAβN for less than 15 min before injection, preliminary assays showed that 1 mM PAβN treatment *in vitro* (for up to 1 h) does not significantly affect *P*. *aeruginosa* cell viability (data not shown). One hundred-µl aliquots of the same suspensions were plated on LB agar to determine the number of viable cells (CFU) injected in the larvae. Larvae were incubated at 30 °C in Petri dishes (ten larvae per dish) and monitored over four days. Larvae were considered dead when they did not respond to gentle prodding. At least 30 larvae were inoculated per condition, in three independent experiments.

### Statistical analysis

Statistical significance was determined by calculating the *p-*values using the two-tailed Student-t test for unpaired data sets; differences with a *p*-value ≤ 0.05 are considered as statistically significant.

## Electronic supplementary material

Supplementary material
