# Supplementary material for: Effect of efflux pump inhibition on Pseudomonas aeruginosa transcriptome and virulence
Source: Sci Rep. 2017 Sep 12;7:11392. doi: 10.1038/s41598-017-11892-9 (PMC5596013; doi:10.1038/s41598-017-11892-9)

Effect of efflux pump inhibition on *Pseudomonas aeruginosa* transcriptome and virulence

Giordano Rampioni[1], Cejoice Ramachandran Pillai[1#], Francesca Longo[1], Roslen Bondì[1], Valerio Baldelli[1], Marco Messina[1], Francesco Imperi[2], Paolo Visca[1], Livia Leoni[1*]

[1]*Department of Science, University Roma Tre, Rome, Italy;* [2]*Department of Biology and Biotechnology "Charles Darwin", Sapienza University of Rome, Rome, Laboratory affiliated to Istituto Pasteur Italia – Fondazione Cenci Bolognetti, Rome, Italy.*

Present address: [#]Inter University Centre for Bioscience, Kannur University, Palayad, Kerala, India.

[*] Corresponding author: Livia Leoni - livia.leoni@uniroma3.it

Supporting Information

Table S1. Genes whose transcription is affected by PAßN

Table S2. Bacterial strains used in this study

Table S3. Oligonucleotides used in this study

Figure S1. Effect of PAßN on *P. aeruginosa* growth

Figure S2. Effect of PAßN on the promoter activity of *lasI* and *lasR* genes

Table S1. Genes whose transcription is affected by PAßN

PA number [a]	Gene name [a]	Fold change [b]	Product name [a]
PA0509[*]	*nirN*	2.27	NirN
PA0510[*]	*nirE*	2.33	NirE
PA0511[*]	*nirJ*	2.23	heme d_1 biosynthesis protein NirJ
PA0514[*]	*nirL*	2.3	heme d_1 biosynthesis protein NirL
PA0516[*]	*nirF*	2.3	heme d_1 biosynthesis protein NirF
PA0517[*]	*nirC*	3.53	probable *c*-type cytochrome precursor
PA0518[*]	*nirM*	3.32	cytochrome c_{551} precursor
PA0519[*]	*nirS*	4.48	nitrite reductase precursor
PA0523[*]	*norC*	2.87	nitric-oxide reductase subunit C
PA0524[*]	*norB*	5.51	nitric-oxide reductase subunit B
PA0525[*]	*norD*	2.19	probable dinitrification protein NorD
PA0526	---	2.54	hypothetical protein
PA0672[ʃ]	*hemO*	-4.81	heme oxygenase
PA0676[ʃ]	*vreR*	-4.85	sigma factor regulator, VreR
PA0688	*lapA*	-3.16	low-molecular-weight alkaline phosphatase A, LapA
PA0697	---	-3.74	hypothetical protein
PA0698	---	-3.03	hypothetical protein
PA0707	*toxR*	-2.12	transcriptional regulator ToxR
PA0842	---	-4.32	probable glycosyl transferase
PA0918	---	2.26	cytochrome b_{561}
PA1116	---	-3.13	hypothetical protein
**PA1168**	---	2.94	hypothetical protein
PA1169	---	2.41	probable lipoxygenase
PA1213	---	2.03	hypothetical protein
**PA1215**	---	2.64	hypothetical protein
**PA1217**	---	2.38	probable 2-isopropylmalate synthase
**PA1221**	---	2.01	hypothetical protein
**PA1245**	*aprX*	-3.44	AprX
PA1300[ʃ]	---	-4.92	ECF sigma factor
PA1301[ʃ]	---	-3.43	probable transmembrane sensor
PA1355[ʃ]	---	-2.99	hypothetical protein
PA1877	---	-2.16	probable secretion protein
**PA1901**[§]	*phzC1*	2.24	phenazine biosynthesis protein PhzC
**PA1902**[§]	*phzD1*	2.26	phenazine biosynthesis protein PhzD
**PA1903**[§]	*phzE1*	2.22	phenazine biosynthesis protein PhzE
**PA1904**[§†]	*phzF1*	2.11	probable phenazine biosynthesis protein
PA1912[ʃ]	*femI*	-2.35	ECF sigma factor, FemI
PA2033	---	-4.72	hypothetical protein
PA2034	---	-3.92	hypothetical protein
**PA2143**	---	-2.2	hypothetical protein
PA2384	---	-4.71	hypothetical protein
PA2385[ʃ]	*pvdQ*	-2.98	$3OC_{12}$-homoserine lactone acylase PvdQ
PA2386[ʃ]	*pvdA*	-3.97	L-ornithine N^5-oxygenase
PA2393	---	-3.09	putative dipeptidase

PA number [a]	Gene name [a]	Fold change [b]	Product name [a]
PA2394[∫]	*pvdN*	-2.85	PvdN
PA2395[∫]	*pvdO*	-2.32	PvdO
PA2396[∫]	*pvdF*	-3.22	pyoverdine synthetase F
PA2397[∫]	*pvdE*	-3.16	pyoverdine biosynthesis protein PvdE
PA2398[∫]	*fpvA*	-2.03	ferripyoverdine receptor
PA2399[∫]	*pvdD*	-3.32	pyoverdine synthetase D
PA2400[∫]	*pvdJ*	-3.36	PvdJ
PA2402[∫]	---	-3.43	probable non-ribosomal peptide synthetase
PA2405	---	-2.42	hypothetical protein
PA2411	---	-4.31	probable thioesterase
PA2412	---	-6.16	conserved hypothetical protein
PA2413[∫]	*pvdH*	-3.58	L-2,4-diaminobutyrate:2-ketoglutarate 4-aminotransferase
PA2424[∫]	*pvdL*	-3.73	PvdL
PA2425[∫]	*pvdG*	-2.58	PvdG
PA2426[∫]	*pvdS*	-10.48	sigma factor PvdS
PA2427	---	-3.62	hypothetical protein
PA2428	---	-18.06	hypothetical protein
**PA2570**	*lecA*	-2.42	LecA
PA2593	*qteE*	2.06	quorum threshold expression element, QteE
**PA3195**	*gapA*	2.14	glyceraldehyde 3-phosphate dehydrogenase
PA3377[◊]	*phnJ*	-21.1	conserved hypothetical protein
PA3392[*]	*nosZ*	2.16	nitrous-oxide reductase precursor
PA3407[∫]	*hasAp*	-4.15	heme acquisition protein HasAp
PA3410	*hasI*	-2.03	HasI
PA3530[∫]	*bfd*	-2.54	bacterioferritin-associated ferredoxin Bfd
PA3581	*glpF*	-2.06	glycerol uptake facilitator protein
PA3586	---	-2.2	probable hydrolase
PA3602	*yerD*	2.07	conserved hypothetical protein
PA3720	---	2.87	hypothetical protein
PA3880	---	2.63	conserved hypothetical protein
PA3899	*fecI*	-2.25	FecI
**PA4139**[†]	---	2.01	hypothetical protein
PA4220	*fptB*	-2.48	hypothetical protein
PA4221[∫]	*fptA*	-2.52	Fe(III)-pyochelin outer membrane receptor precursor
PA4224[∫]	*pchG*	-2.05	pyochelin biosynthetic protein PchG
PA4225[∫]	*pchF*	-2.26	pyochelin synthetase
PA4226[∫]	*pchE*	-2.06	dihydroaeruginoic acid synthetase
PA4228[∫]	*pchD*	-2.09	pyochelin biosynthesis protein PchD
PA4230[∫]	*pchB*	-2.69	salicylate biosynthesis protein PchB
PA4333	*fumA*	2.01	probable fumarase
PA4430	---	2.28	probable cytochrome *b*
PA4467	---	-3.7	hypothetical protein
PA4468	*sodM*	-11.65	superoxide dismutase
PA4469	---	-10.96	hypothetical protein
PA4470	*fumC1*	-8.96	fumarate hydratase
PA4471	*fagA*	-7.96	hypothetical protein

**PA number** [a]	**Gene name** [a]	**Fold change** [b]	**Product name** [a]
PA4504	*dppC*	2.18	probable permease of ABC transporter
PA4515	*piuC*	-2.48	conserved hypothetical protein
PA4570	---	-5.53	hypothetical protein
PA4587	*ccpR*	3.2	cytochrome c_{551} peroxidase precursor
**PA4703**	---	-2.04	hypothetical protein
PA4708[∫]	*phuT*	-3.17	heme-transport protein, PhuT
PA4709[∫]	*phuS*	-3.37	PhuS
PA4710[∫]	*phuR*	-4.65	meme/memoglobin uptake outer membrane receptor PhuR
PA4810[*]	*fdnI*	2.22	nitrate-inducible formate dehydrogenase, γ subunit
PA5091	*hutG*	2.11	*N*-formylglutamate amidohydrolase
PA5098	*hutH*	3.16	histidine ammonia-lyase
PA5100	*hutU*	2.95	urocanase
PA5360[◊]	*phoB*	-15.28	two-component response regulator PhoB
PA5365[◊]	*phoU*	-9.4	phosphate uptake regulatory protein PhoU
PA5366[◊]	*pstB*	-14.02	ATP-binding component of ABC phosphate transporter
PA5367[◊]	*pstA*	-14.17	membrane protein component of ABC phosphate transporter
PA5369[◊]	*pstS*	-23.49	periplasmic phosphate-binding protein, PstS
PA5410	*gbcA*	2.14	GbcA

[a] PA number, gene name and product name are from the *Pseudomonas* Genome Database[23]. Genes previously reported as controlled by $3OC_{12}$-HSL are in bold characters[36-38]. [*], genes involved in nitrogen metabolism; §, genes involved in phenazines biosynthesis; [∫], genes previously reported to be controlled by iron-starvation[25]; [◊], genes previously reported to be controlled by phosphate-starvation[27]; [†], genes previously reported to be controlled by colistin[31].

[b] Fold change in gene expression in *P. aeruginosa* PAO1 grown in LB supplemented with 27 µM PAßN with respect to the same strain grown in LB.

Table S2. Bacterial strains used in this study

Strains	Relevant characteristics	Reference/Source
P. aeruginosa		
PAO1	PAO1 wild type strain ATCC15692	ATCC
PAO1-KP	PAO1 wild type strain gently provided by Prof. K. Poole, Queen's University, Kingston, Canada	[34]
PAO1-KP Δefflux	PAO1-KP mutant strain carrying mutations in the efflux pumps MexAB-OprM, MexCD-OprJ, MexEF-OprN and MexXY-OprM. Gently provided by Prof. K. Poole, Queen's University, Kingston, Canada	[34]
PA14-R3	biosensor strain for $3OC_{12}$-HSL quantification	[51]
PAO1 Δ*pqsAH PpqsA::lux*	biosensor strain for HHQ/PQS quantification	[54]
PAO-JP2 pKD-*rhlA*	biosensor strain for C_4-HSL quantification	[55]

Additional references for Table S1:

54. Fletcher, M. P. *et al.* A dual biosensor for 2-alkyl-4-quinolone quorum-sensing signal molecules. *Environ. Microbiol.* **9,** 2683-2693 (2007).

55. Duan, K. & Surette, M. G. Environmental regulation of *Pseudomonas aeruginosa* PAO1 Las and Rhl quorum-sensing systems. *J. Bacteriol.***189,** 4827-4836 (2007).

Table S3. Oligonucleotides used in this study

Name	Sequence (5'-3') [a]	Restriction site [b]
FW*pqsB*	CCGCTCGAGCGACCAGGGCTATCGCA	XhoI
RV*pqsB*	CCGGAATTCCTTATGCATGAGCTTCTCC	EcoRI
FW16SRT	AGTACGGCCGCAAGGTTAAA	-
RV16SRT	CCCAACATCTCACGACACGA	-
FW*aprX*RT	CTGCCGATCAACGTCTCCTT	-
RV*aprX*RT	TACCGTAGAACTTGGCGCTG	-
FW*sodM*RT	CGTTCAAGGATGCGTTCACC	-
RV*sodM*RT	GCGGTTCTGGTACTTCAGGT	-
FW*norB*RT	TTCCTGTTCAACGTCGGCAT	-
RV*norB*RT	GCACCCATGATCAGTTCCCA	-
FW*qteE*RT	GATGCGGTGAGCGACTACAT	-
RV*qteE*RT	GAAGATGCTGGTTGGCATCG	-
FW*pvdQ*RT	GAAGACGCTCGAGGAGATGG	-
RV*pvdQ*RT	TGAAGCGCTGGAAGTAGACG	-
FW*pvdS*RT	GGAACAACTGTCTACCCGCA	-
RV*pvdS*RT	GTAGCTGAGCTGTGCCTTGA	-
FW*fumC1*RT	GAACTGAACGTGATGCTGCC	-
RV*fumC1*RT	TTTCCGCAGCCTTCTGGTAG	-

[a] Engineered restriction sites are underlined.

[b] -, no restriction site introduced.

Figure S1. Effect of PAßN on *P. aeruginosa* growth

Growth of *P. aeruginosa* PAO1 in LB in the absence or in the presence of the indicated PAßN concentrations. The average of at least three independent experiments is reported with SD.

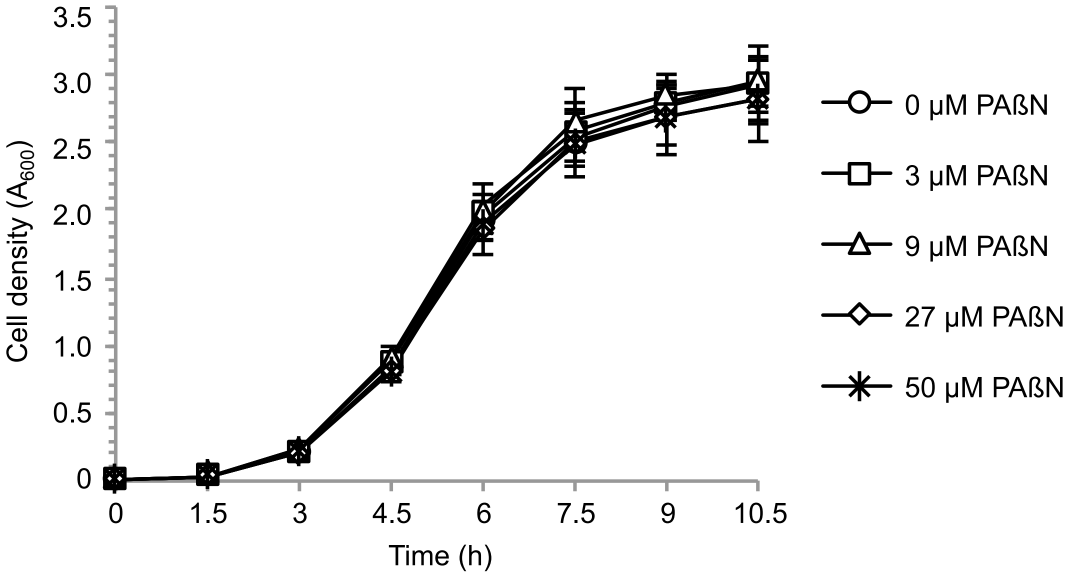

Figure S2. Effect of PAßN on the promoter activity of *lasI* and *lasR* genes

P*lasI* and P*lasR* promoter activity measured in the *P. aeruginosa* PAO1 strain carrying a chromosomally inserted cassettes P*lasI::lux* or P*lasR::lux*, respectively. The strains were grown at 37ºC with 120 rpm shaking in LB in the absence or in the presence of 27 µM PAßN. Promoter activity is reported as Relative Light Units (RLUs) normalized to bacterial cell density (A_{600}). The average of three independent experiments is reported with SD.

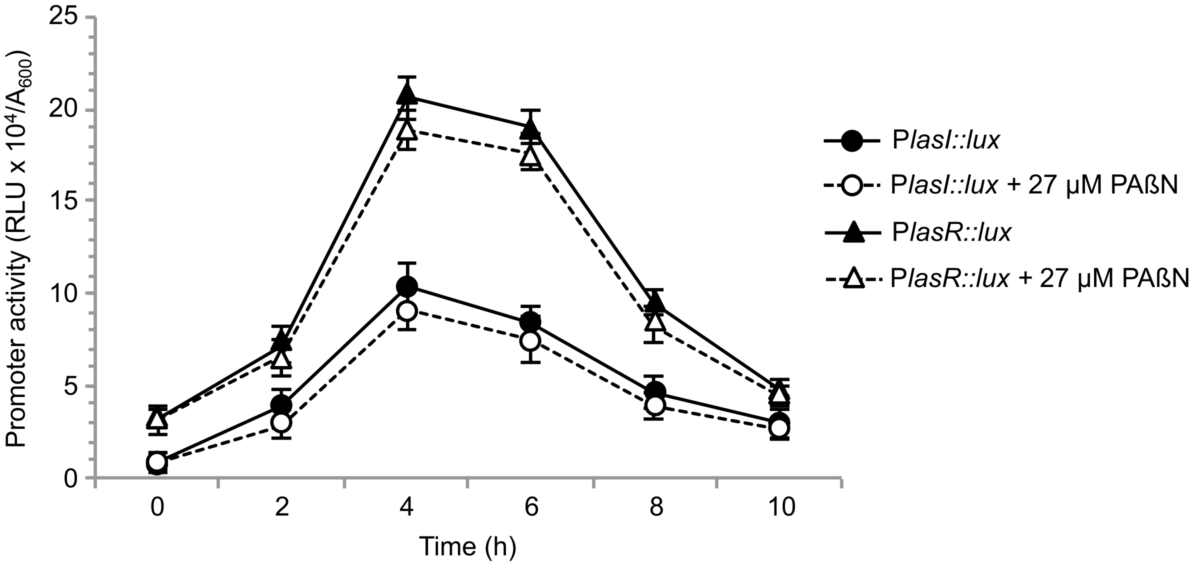

Supplement: Supplementary file 1 — Supplementary material [file 41598_2017_11892_MOESM1_ESM.pdf]
